# Reduced contrast sensitivity function correlated with superficial retinal capillary plexus impairment in early stage of dysthyroid optic neuropathy

**DOI:** 10.1186/s40662-023-00328-3

**Published:** 2023-02-04

**Authors:** Yunhai Tu, Haochen Jin, Mingna Xu, Weijie Liu, Xiaozhou Hu, Mengting Wang, Jie Ye, Zihui Liu, Mengyuan Gao, Fang Hou, Zhong-Lin Lu, Wencan Wu

**Affiliations:** 1 The Eye Hospital, School of Ophthalmology & Optometry, Wenzhou Medical University, Wenzhou, 325027 China; 2 Oujiang Laboratory (Zhejiang Lab for Regenerative Medicine, Vision and Brain Health), Wenzhou, 325000 Zhejiang China; 3 Division of Arts and Sciences, NYU Shanghai, Shanghai, China; 4 Center for Neural Science and Department of Psychology, New York University, New York, USA; 5 NYU-ECNU Institute of Brain and Cognitive Science, NYU Shanghai, Shanghai, China

**Keywords:** Contrast sensitivity function, Visual impairments, Superficial retinal capillary plexus, Thyroid-associated ophthalmopathy, Dysthyroid optic neuropathy

## Abstract

**Background:**

To assess the accuracy of contrast sensitivity function (CSF) in detecting dysthyroid optic neuropathy (DON) at an early stage in thyroid-associated ophthalmopathy (TAO) patients and to examine potential factors that may be linked to early visual impairments in these individuals.

**Methods:**

A total of 81 TAO patients (50 non-DON and 31 DON), and 24 control subjects participated in the study. CSF was measured with the quick CSF (qCSF) method. Optical coherence tomography angiography (OCTA) images of the ganglion cell complex layer (GCCL), superficial and deep retinal capillary plexuses (SRCP and DRCP) in a 3 mm diameter area around the macula were evaluated.

**Results:**

Compared with the controls, the area under the log contrast sensitivity function (AULCSF) and SRCP density were significantly reduced in non-DON and DON patients (all *P* < 0.05). The GCCL thickness of the DON patients was thinner than that of the controls and non-DON patients (all *P* < 0.05). The AULCSF was significantly correlated with spherical equivalent refractive error, muscle index, SRCP density and GCCL thickness in TAO patients, respectively (all *P* < 0.05). However, stepwise multi-regression analysis showed that the AULCSF was only significantly correlated with SRCP density (*P* < 0.001). Receiver operating characteristic curve analysis showed that the AULCSF produced the most accurate discrimination between non-DON and DON patients from the controls (AUC = 0.831, 0.987, respectively; all *P* < 0.001).

**Conclusions:**

CSF change in the early stage of DON is related to SRCP density. It can be an early indicator of visual impairments associated with DON in TAO patients.

## Background

Thyroid-associated ophthalmopathy (TAO), a complication of Graves’ hyperthyroidism, is an autoimmune disorder that affects the orbital and periorbital tissues [1]. 5–8% of TAO patients have dysthyroid optic neuropathy (DON) [2], a severe complication characterized by a range of visual impairments including reduced visual acuity (VA), visual field (VF) deficits, abnormal color vision, relative afferent pupillary deficits in unilateral cases, and reduced contrast sensitivity function (CSF) [3]. If left untreated, these patients may experience permanent visual impairments [4]. Early detection of DON could potentially allow earlier intervention and prevent visual impairments.

VA and VF tests are commonly used to evaluate visual impairments of DON in clinical settings. However, McKeag et al. [5] reported that VA may not be a reliable indicator of DON. Labonia et al. [6] found that the high prevalence of VF loss in TAO patients may be due to a high rate of false positive results. These reports suggest that VA and VF tests may not be suitable for accurate detection of visual impairments in early DON.

CSF, on the other hand, provides a comprehensive evaluation of spatial vision at various spatial frequencies [7] and is better correlated with daily visual function in tasks such as reading, driving, and walking [8]. It is more sensitive in detecting subtle functional differences in early retinopathy and optic neuropathy [9–12]. Previous studies have shown that CSF is reduced in moderate to severe DON patients who experienced significant orbital apex crowding and significant vision loss [13–16]. However, CSF change in TAO patients without clinical signs of DON has not been reported. The aim of this study was to investigate the accuracy of CSF in early detection of DON.

Mechanical compression caused by orbital apex crowding is thought to be a major contributor to visual impairments in TAO patients. However, this theory does not explain all visual impairments in TAO patients [17, 18]. Using high resolution optical coherence tomography (OCT) and OCT angiography (OCTA), our previous study found that the thickness of the inner intra-retinal layer and the density of microvasculature around the macula were significantly reduced in TAO patients without evident apical crowding. There was also a significant relationship between the macular inner retinal thickness, microvascular density, and VA [19]. We and others [20–22] have also reported that reduced peripapillary vessel density is significantly correlated with VF loss. We hypothesized that there could be factors other than mechanical compression such as retinal microvasculature and structure that could contribute to the visual impairments in early DON.

Here, we systematically measured CSF, inner retinal microvascular density and thickness around the macula in TAO patients, especially in those with normal VA, VF, optic nerve head appearance, and no obvious evidence of orbital apex crowding. We then examined the relationships between CSF and clinical manifestations, orbital apex crowding, and retinal microvasculature and structure. Through detailed analysis, we aimed to identify the best indicator of early DON.

## Methods

### Subjects

This cross-sectional study was approved by the ethics committee of the Affiliated Eye Hospital of Wenzhou Medical University, Zhejiang, China (No. 2020-142-K-127). All subjects provided written informed consent and the study adhered to the Declaration of Helsinki. A total of 81 patients with TAO participated in the study, including 50 without DON (non-DON) and 31 with DON. The study took place between February 2021 and September 2021 at the Affiliated Eye Hospital of Wenzhou Medical University. Control subjects were recruited simultaneously and matched for age and sex. The diagnosis of TAO and clinical activity score (CAS) were generated according to the European Group on Graves’ Orbitopathy criteria [23]. DON was diagnosed based on decreased best-corrected VA (BCVA, > 0 logMAR), VF loss with a mean deviation (MD) less than − 2 dB, and optic disk edema. Patients with TAO who had normal BCVA (≤ 0 logMAR), normal VF (MD ≥ − 2 dB), and no signs of optic disk edema were placed in the non-DON group. Eyes with severe optic nerve involvement (i.e., significant impairment of BCVA [> 0.3 logMAR], evident apical crowding in orbital computed tomography [CT], and appearance of relative afferent pupillary deficits in unilateral cases), spherical equivalent refractive error (SE) over + 1.50 diopters (D) or under − 4.00 D, astigmatism over − 1.50 D, significant cataracts, corneal involvement, glaucoma, retinal diseases or vasculitis, or systemic and autoimmune diseases other than thyroid disorders were excluded from the study.

### Clinical examinations

All patients underwent a series of ophthalmological examinations, including refraction, BCVA test, slit-lamp biomicroscopy, exophthalmometry measurements and orbital CT. We calculated the muscle index (MI) according to the method described by Barrett et al. [24], and evaluated the maximum medial rectus (MR) diameter using axial scans as described previously [25]. Fundus photographs were taken with a 45-degree digital retinal camera (Visucam 224; Carl Zeiss Meditec, Inc., Dublin, CA, USA). Perimetry was conducted using the 30-2 strategy (Humphrey Field Analyzer II; Carl Zeiss Meditec, Inc., Dublin, CA, USA), and non-contact intraocular pressure (IOP) was measured by a Full Auto Tonometer TX-20 (Topcon, Tokyo, Japan). Control subjects underwent the same examinations as the TAO patients, except for exophthalmometry measurements and orbital CT.

### Contrast sensitivity function test

The CSF was measured using the Manifold Contrast Vision Meter (AST Inc., San Diego, CA). The device incorporated the quick CSF (qCSF) method [26, 27] to efficiently estimate the entire CSF curve, which has been applied in quantifying functional vision deficits in many clinical conditions [9–12].

All subjects wore the best-correcting spectacles and were tested monocularly, with the untested eye covered with a patch. The duration for this test was approximately 5 min. Before the test, subjects were dark adapted for 5 min, during which they were given demo trials to become familiar with the experimental procedure. Contrast sensitivity at 1.0, 1.5, 3.0, 6.0, 12.0 and 18.0 cycles per degree (cpd) and the area under the log contrast sensitivity function (AULCSF) from 1.0 to 18.0 cpd were reported by the Manifold Contrast Vision Meter.

### Optical coherence tomography angiography

All subjects were imaged with an OCTA system (Optovue RTVue XR Avanti; Optovue, Inc., Fremont, CA, USA). The size of OCTA images was 304 × 304 pixels and the scan area, captured by combining the Fx and Fy scans centered on the fovea, was 3 × 3 mm^2^. The ganglion cell complex layer (GCCL), superficial and deep retinal capillary plexuses (SRCP and DRCP) were detected and separated automatically by the OCTA instrument. Retinal vessel densities and thickness in the macular region with a diameter of 3.0 mm were automatically calculated using the device’s program. Images with signal strength index less than 6/10, poor scan centering, and motion artifacts were excluded.

### Statistical analyses

All data were analyzed with SPSS (version 26.0; IBM Corp., Armonk, NY, USA). The sex differences of the three groups were determined by the Chi-squared test. One-way analysis of variance (ANOVA) was used to compare the differences among the three groups, and post hoc tests were used for between group comparisons. Univariate regression and stepwise multi-regression analyses were used to explore the factors that might be associated with AULCSF. The receiver operating characteristic (ROC) curve analysis was conducted to evaluate the classification accuracy in discriminating non-DON and DON patients from the controls based on AULCSF, inner retinal microvascular density and thickness. In addition, we calculated classification accuracy based on multiple indexes through the multivariate logistic regression model [28]. Larger areas under the ROC curve (AUC) indicated higher diagnostic values. *P* values less than 0.05 were considered statistically significant.

## Results

### Basic patient characteristics

The demographic characteristics, AULCSF and OCTA parameters of the subjects are shown in Table 1. There were no significant differences in age, sex or SE among the control, non-DON and DON groups (all *P* > 0.05). BCVA, MD and IOP differed significantly across the three groups (all *P* < 0.05). The non-DON and control groups did not differ significantly in BCVA and MD (all *P* > 0.05), and the DON and non-DON groups did not differ significantly in exophthalmometry, CAS, MI and maximal MR diameter (all *P* > 0.05).

**Table 1:** Demographic characteristics, AULCSF and OCTA parameters of the subjects

Parameters	Control	non-DON	DON	*P* value*	*P* value_1_	*P* value_2_	*P* value*3*
N	24	50	31	–	–	–	–
Age (years)	45.2 ± 7.1	45.7 ± 8.8	48.1 ± 9.8	0.404	0.808	0.234	0.249
Sex (female/male)	17/7	32/18	22/9	0.752	0.561	0.991	0.518
SE (D)	− 0.86 ± 1.48	− 0.59 ± 1.41	− 0.17 ± 1.05	0.151	0.839	0.167	0.342
IOP (mmHg)	14.28 ± 1.58	16.70 ± 3.57	16.77 ± 3.85	0.008	0.001	0.006	1.000
Exophthalmometry (mm)	–	17.81 ± 3.10	18.85 ± 3.50	–	–	–	0.169
CAS	–	1.08 ± 0.85	1.43 ± 0.90	–	–	–	0.083
MI	–	0.60 ± 0.10	0.64 ± 0.11	–	–	–	0.146
Maximal MR diameter (mm)	–	6.51 ± 2.66	7.06 ± 2.13	–	–	–	0.388
BCVA (logMAR)	− 0.01 ± 0.03	− 0.00 ± 0.02	0.15 ± 0.07	< 0.001	0.598	< 0.001	< 0.001
MD (dB)	− 0.95 ± 1.15	− 1.10 ± 1.20	− 5.26 ± 3.81	< 0.001	0.935	< 0.001	< 0.001
AULCSF	1.31 ± 0.10	1.13 ± 0.15	0.88 ± 0.18	< 0.001	< 0.001	< 0.001	< 0.001
SRCP (%)	46.59 ± 1.72	45.01 ± 2.65	41.90 ± 3.35	< 0.001	0.009	< 0.001	< 0.001
DRCP (%)	50.07 ± 2.54	49.21 ± 3.01	48.74 ± 3.46	0.276	0.261	0.112	0.499
GCCL (μm)	103.55 ± 6.77	104.04 ± 7.41	97.53 ± 10.20	0.002	0.808	0.008	0.001

Data are shown as mean ± standard deviation in each group

*Control* = control eyes; *DON* = dysthyroid optic neuropathy; *non-DON* = TAO patients without clinical signs of DON; *TAO* = thyroid-associated ophthalmopathy; *SE* = spherical equivalent refractive error; *IOP* = intraocular pressure; *CAS* = clinical activity score; *MI* = muscle index; *MR* = medial rectus; *BCVA* = best-corrected visual acuity; *MD* = mean deviation of visual field; *AULCSF* = area under the log contrast sensitivity function; *SRCP* = superficial retinal capillary plexus; *DRCP* = deep retinal capillary plexus; *GCCL* = ganglion cell complex layer; – = not performed

*P* value* = *P* value among the three groups; *P* value_1_ = *P* value between the control and non-DON groups; *P* value_2_ = *P* value between the control and DON groups; *P* value_3_ = *P* value between the non-DON and DON groups

### Contrast sensitivity function

**Fig. 1:**
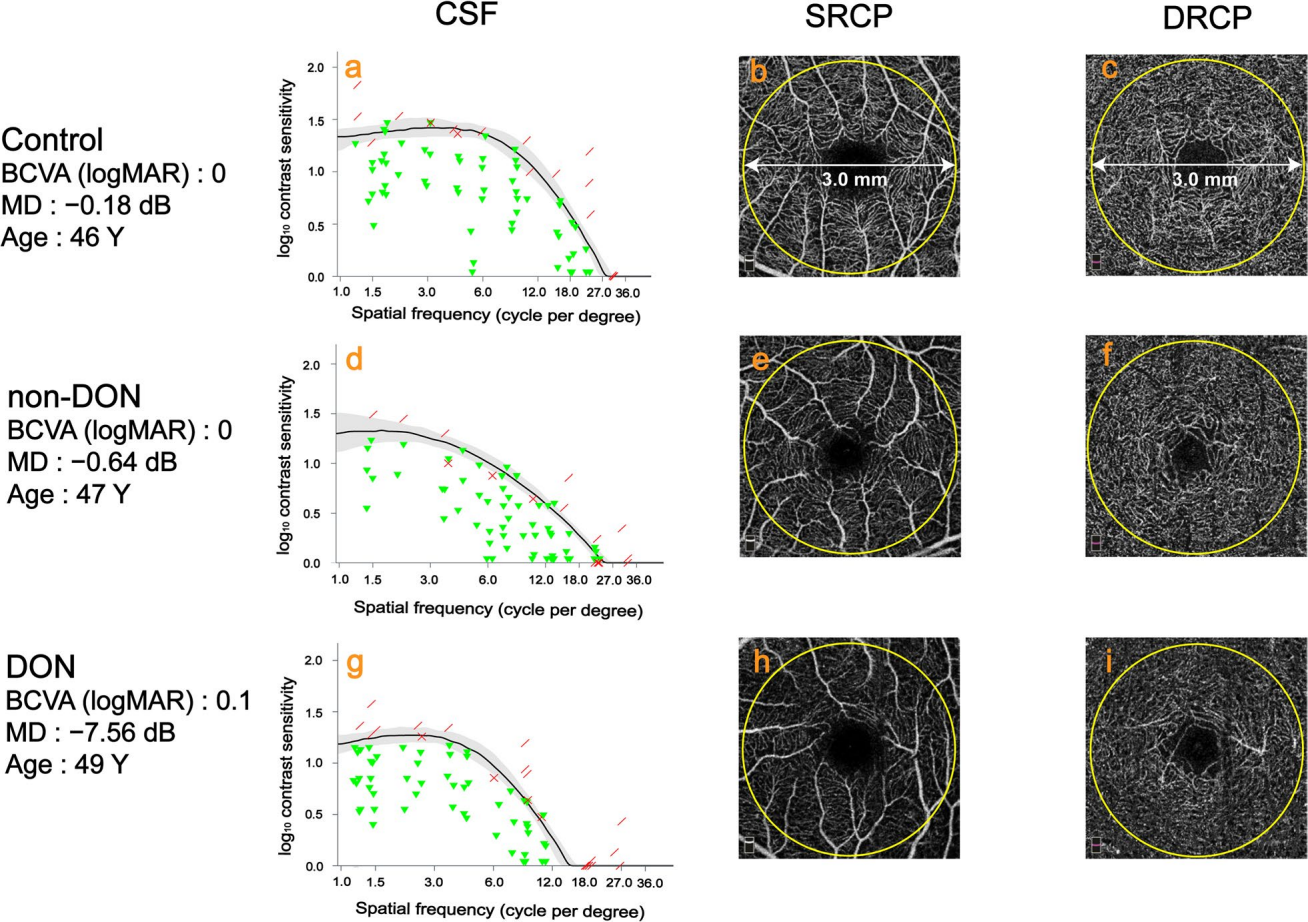
Representative CSF test results and OCTA images of control subjects, TAO patients without clinical signs of DON (non-DON), and TAO patients with DON (DON). **a**, **d**, **g** CSF test results; **b**, **e**, **h** OCTA images of the SRCP; **c**, **f**, **i** OCTA images of the DRCP. CSF, contrast sensitivity function; OCTA, optical coherence tomography angiography; TAO, thyroid-associated ophthalmopathy; DON, dysthyroid optic neuropathy; SRCP, superficial retinal capillary plexus; DRCP, deep retinal capillary plexus; Y, years

**Fig. 2:**
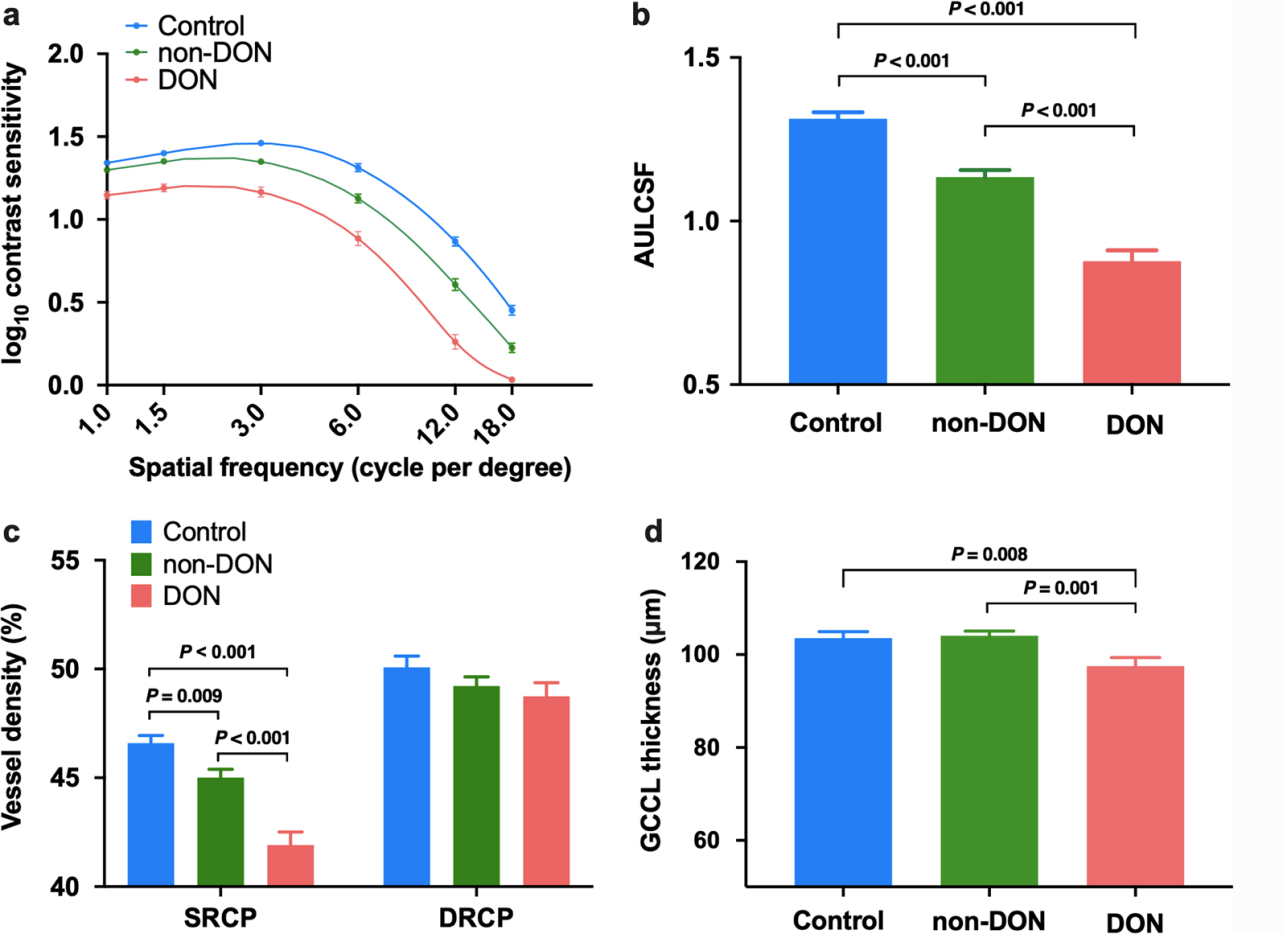
CSF, inner retinal microvascular density and GCCL thickness among the three groups. **a** Contrast sensitivity curves over 1.0, 1.5, 3.0, 6.0, 12.0 and 18.0 cpd; **b** Area under the log contrast sensitivity function (AULCSF); **c** Vessel densities of SRCP and DRCP; **d** GCCL thickness. Error bars represent ± 1 standard error of the mean. The colors blue, green and red represent the control, non-DON and DON groups, respectively. CSF, contrast sensitivity function; GCCL, ganglion cell complex layer; SRCP, superficial retinal capillary plexus; DRCP, deep retinal capillary plexus; DON, dysthyroid optic neuropathy; non-DON, TAO patients without clinical signs of DON; TAO, thyroid-associated ophthalmopathy

CSF of one representative subject from each of the three groups is shown in Fig. 1a, d and g. Figure 2a depicts the contrast sensitivity curves over 1.0, 1.5, 3.0, 6.0, 12.0 and 18.0 cpd. The CSF of the non-DON group was not significantly different from that of the control group at 1.0 and 1.5 cpd (*P* > 0.05), but significantly lower than that of the control group at 3.0, 6.0, 12.0 and 18.0 cpd (*P* < 0.001). The CSF of the DON group was significantly lower than that of the control and non-DON groups across all spatial frequencies (all *P* < 0.001). As revealed by one-way ANOVA analysis, there was significant difference in AULCSF among the three groups (*P* < 0.001, Table 1, Fig. 2b). In addition, the AULCSF of the control group was greater than that of the non-DON and DON groups (all *P* < 0.001, Table 1, Fig. 2b).

### Inner retinal microvascular density, GCCL thickness and their relationships

**Fig. 3:**
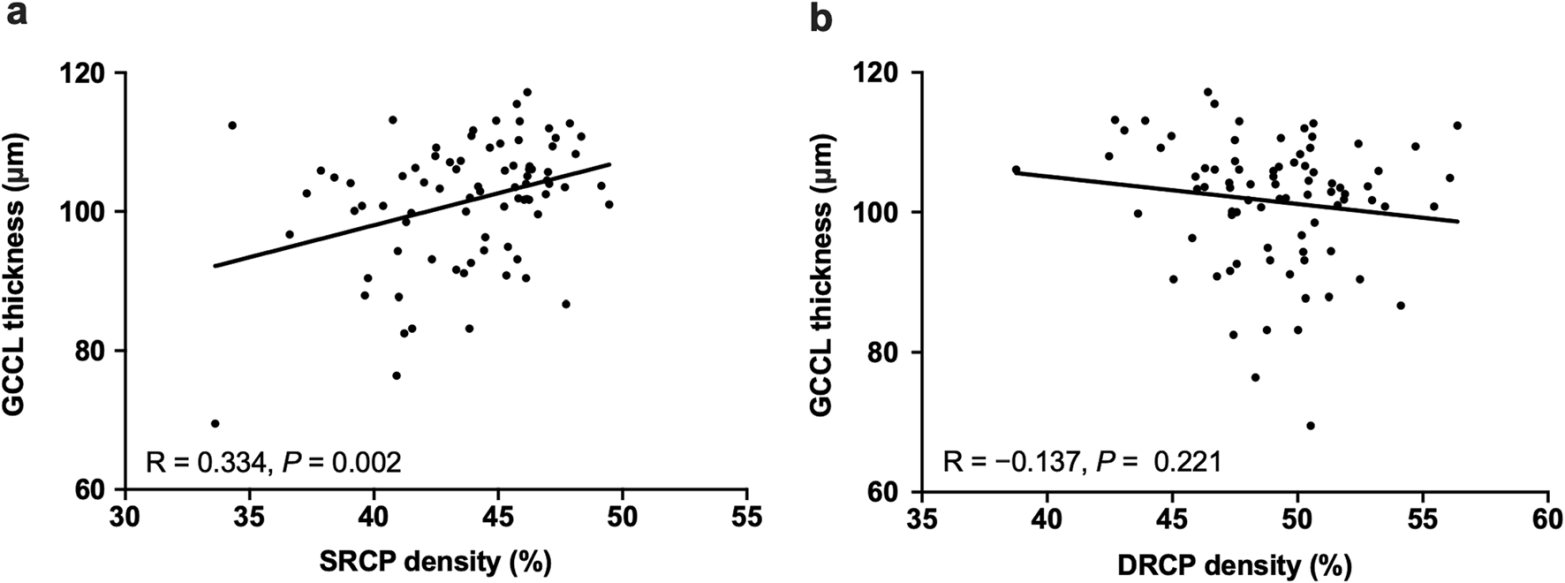
Correlation between GCCL thickness and inner retinal microvascular density in TAO patients. **a** Correlation between GCCL thickness and SRCP density; **b** Correlation between GCCL thickness and DRCP density. GCCL, ganglion cell complex layer; TAO, thyroid-associated ophthalmopathy; SRCP, superficial retinal capillary plexus; DRCP, deep retinal capillary plexus

OCTA images of the superficial and deep retinal capillary layers of representative subjects in the control, non-DON and DON groups are shown in Fig. 1. While there was no significant difference in DRCP density among the three groups (*P* = 0.276, Table 1, Fig. 2c), a significant SRCP density difference was found (*P* < 0.001, Table 1, Fig. 2c). Additionally, SRCP density of the control group (46.59 ± 1.72%) was significantly higher than that of the non-DON (45.01 ± 2.65%) and DON (41.90 ± 3.35%) groups (all *P* < 0.05). Although there was no significant difference of GCCL thickness between the control and non-DON groups (*P* = 0.808), GCCL thickness of the DON group was significantly thinner than that of the control (*P* = 0.008) and non-DON (*P* = 0.001) groups (Table 1, Fig. 2d). Moreover, GCCL thickness was positively correlated with SRCP density (R = 0.334, *P* = 0.002, Fig. 3a), but not correlated with DRCP density (R =  − 0.137, *P* = 0.221, Fig. 3b) in TAO patients.

### Factors underlying decreased AULCSF in patients with TAO

Table 2 summarizes correlation coefficients between AULCSF and several other independent variables in the patients with TAO, including age, SE, IOP, exophthalmometry, CAS, MI, maximal MR diameter, SRCP and DRCP densities, and GCCL thickness. In the univariate regression analysis, SE, MI, SRCP density and GCCL thickness were significantly correlated with decreased AULCSF (all *P* < 0.05). The stepwise multi-regression analysis showed that reduced SRCP density was the most important factor related to AULCSF loss (*P* < 0.001).

**Table 2:** Linear regression based on the AULCSF criteria as a dependent factor for TAO patients

Parameters	Univariate regression analysis			Multivariate regression analysis		
	Unstandardized coefficients	Standardized coefficients	*P* value	Unstandardized coefficients	Standardized coefficients	*P* value
Age (years)	− 0.003	− 0.128	0.255	–	–	–
SE (D)	− 0.036	− 0.223	0.045	–	–	–
IOP (mmHg)	− 0.007	− 0.125	0.274	–	–	–
Exophthalmometry (mm)	− 0.003	− 0.043	0.708	–	–	–
CAS	− 0.045	− 0.194	0.085	–	–	–
MI	− 0.645	− 0.334	0.012	–	–	–
Maximal MR diameter (mm)	− 0.014	− 0.166	0.194	–	–	–
SRCP (%)	0.032	0.512	< 0.001	0.032	0.512	< 0.001
DRCP (%)	− 0.003	− 0.042	0.711	–	–	–
GCCL (μm)	0.007	0.287	0.009	–	–	–

*AULCSF* = area under the log contrast sensitivity function; *SE* = spherical equivalent refractive error; *IOP* = intraocular pressure; *CAS* = clinical activity score; *MI* = muscle index; *MR* = medial rectus; *SRCP* = superficial retinal capillary plexus; *DRCP* = deep retinal capillary plexus; *GCCL* = ganglion cell complex layer; *TAO* = thyroid-associated ophthalmopathy; – = not performed

**Fig. 4:**
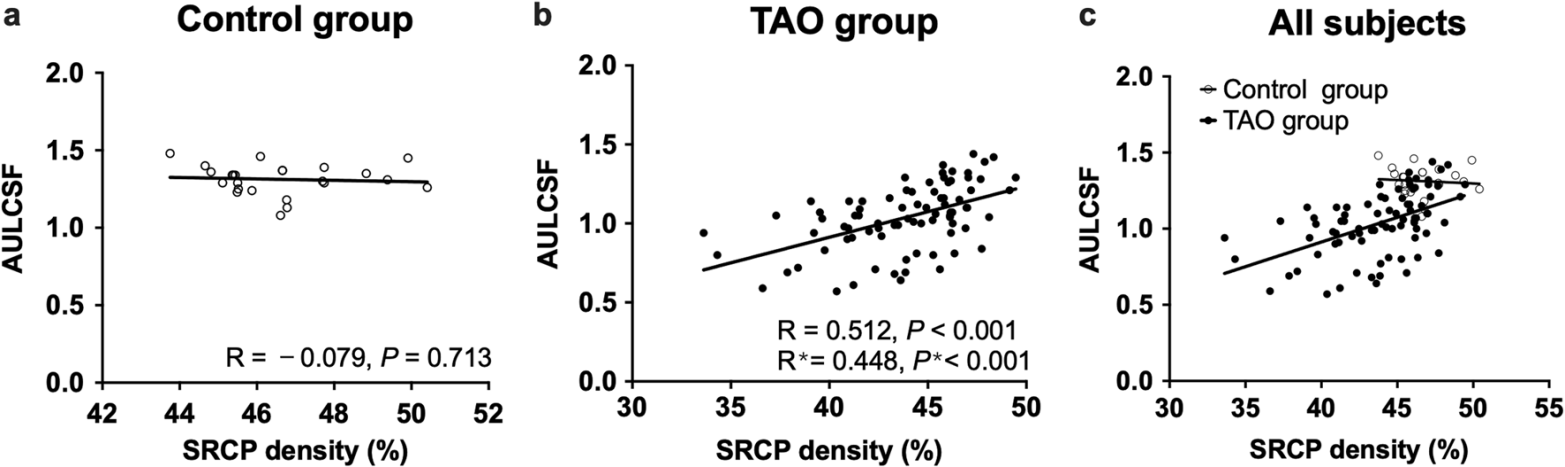
Relationship between the AULCSF and SRCP density in the control (**a**) and TAO (**b**) groups. **c** Scatterplot for SRCP density vs. AULCSF in all subjects. *Indicates *P* value after adjusting the SE, GCCL thickness and MI. AULCSF, area under the log contrast sensitivity function; SRCP, superficial retinal capillary plexus; TAO, thyroid-associated ophthalmopathy SE, spherical equivalent refractive error; GCCL, ganglion cell complex layer; MI, muscle index

We further investigated the relationship between the AULCSF and SRCP density in the control and TAO groups. For control subjects, there was no significant correlation between the AULCSF and SRCP density (R =  − 0.079, *P* = 0.713, Fig. 4a). However, the AULCSF of the TAO patients was significantly correlated with SRCP density, with or without adjustment for SE, GCCL thickness and MI (R = 0.512, 0.448, respectively, all *P* < 0.001, Fig. 4b). A scatter diagram of SRCP density vs. AULCSF for all subjects is shown in Fig. 4c.

### ROC curve analysis

**Fig. 5:**
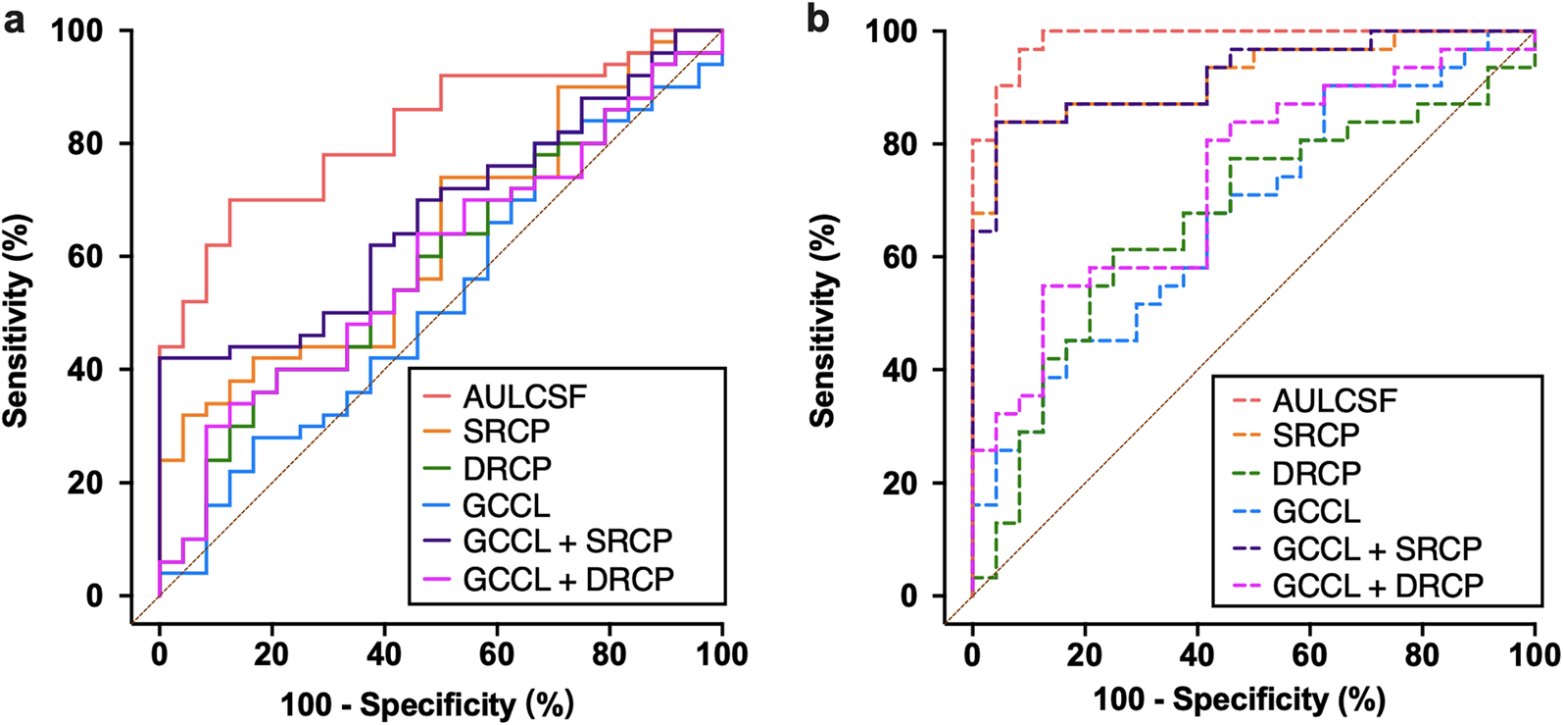
ROC curves of the AULCSF and OCTA parameters in the non-DON and DON patients. **a** ROC curves for non-DON patients vs. controls were generated based on six classification indicators: AULCSF, SRCP density, DRCP density, GCCL thickness, a combination of GCCL thickness and SRCP density, and a combination of GCCL thickness and DRCP density. **b** ROC curves for DON patients vs. controls were generated based on six classification indicators: AULCSF, SRCP density, DRCP density, GCCL thickness, a combination of GCCL thickness and SRCP density, and a combination of GCCL thickness and DRCP density. ROC, receiver operating characteristic; AULCSF, area under the log contrast sensitivity function; OCTA, optical coherence tomography angiography; non-DON, TAO patients without clinical signs of DON; DON, dysthyroid optic neuropathy; TAO, thyroid-associated ophthalmopathy; SRCP, superficial retinal capillary plexus; DRCP, deep retinal capillary plexus; GCCL, ganglion cell complex layer

We performed ROC curve analysis to determine the accuracy of using AULCSF, inner retinal microvascular density and GCCL thickness to distinguish TAO patients with or without clinical signs of DON from the controls. The AUCs of the inner retinal microvascular density and GCCL thickness ranged from 0.473 to 0.639 for non-DON patients, and from 0.667 to 0.921 for DON patients. After combining the indicators of GCCL thickness with the SRCP or DRCP density, the AUCs of the composite index ranged from 0.590 to 0.676 for non-DON patients, and from 0.734 to 0.922 for DON patients. Classifications based on AULCSF were more accurate than those based on inner retinal microvascular density, GCCL thickness and the composite index for non-DON patients (AUC = 0.831, sensitivity = 87.5%, specificity = 70.0%, *P* < 0.001; Fig. 5a; Table 3) and DON patients (AUC = 0.987, sensitivity = 91.7%, specificity = 88.4%, *P* < 0.001; Fig. 5b; Table 3). Although combining the GCCL thickness and inner retinal microvascular density slightly increased classification accuracy, AULCSF remained the best predictor of subject categories.

**Table 3:** Results from ROC curve analysis of patients vs. controls classification based on AULCSF, inner retinal microvascular density and GCCL thickness

Parameters	non-DON				DON			
	AUC	Cutoff	Sensitivity (%)	Specificity (%)	AUC	Cutoff	Sensitivity (%)	Specificity (%)
AULCSF	0.831	1.22	87.5	70.0	0.987	1.17	91.7	88.4
SRCP density (%)	0.639	45.10	87.5	38.0	0.921	44.57	95.8	83.9
DRCP density (%)	0.585	48.91	79.2	40.0	0.667	49.04	75.0	61.3
GCCL thickness (μm)	0.473	100.50	66.7	20.0	0.673	97.70	83.3	45.2

*DON* = dysthyroid optic neuropathy; *non-DON* = TAO patients without clinical signs of DON; *TAO* = thyroid-associated ophthalmopathy; *AULCSF* = area under the log contrast sensitivity function; *SRCP* = superficial retinal capillary plexus; *DRCP* = deep retinal capillary plexus; *GCCL* = ganglion cell complex layer*; ROC* = receiver operating characteristic; *AUC* = areas under the ROC curve

## Discussion

In this study, we found that the CSF of patients with TAO decreased significantly, even in those without visible signs of DON. This indicates that a decrease in CSF is important for detecting DON in its early stage. Additionally, we found that the loss of CSF was significantly correlated with reduced density of the SRCP in TAO patients. Furthermore, ROC curve analysis showed that the AULCSF had significantly higher accuracy in distinguishing non-DON and DON patients from control subjects compared to indicators such as inner retinal microvascular density, GCCL thickness, and their combination.

Most of the previous studies focused on CSF change in moderate to severe DON patients who experienced significant orbital apex crowding and obvious vision loss [13–16], but very few have evaluated the CSF of non-DON patients who have normal VA, VF, optic nerve head appearance, and CT scan results. In our study, we used a new CSF testing device and recruited a group of TAO patients without clinical signs of DON. The device uses a computerized Bayesian active learning CSF test procedure [26], which provides higher test precision and efficiency, and it is more sensitive to small CSF changes [29]. Our results showed patients with normal VA and VF had a significant decrease in CSF, suggesting that the CSF obtained from the Manifold Contrast Vision Meter was more sensitive than VA and VF in detecting subtle functional differences of TAO patients. We suggest that the CSF may serve as a potential functional vision endpoint of early visual impairments in TAO patients. In addition, we found there was a dramatic decrease in the CSF in the DON group compared to the non-DON group, indicating that the CSF reduction may be associated with the severity of the disease.

Previous papers have reported that decreased CSF is correlated with retinal structural and microvascular alterations in patients with high myopia [30], diabetic retinopathy [9] and glaucoma [31]. To investigate the potential factors that may be associated with the CSF change in TAO patients, we also evaluated the retinal microvasculature and structure around the macula by OCTA. Here, we found that SRCP density was significantly decreased in the non-DON and DON groups, whereas no significant DRCP density difference was found among the three groups. Similar results were also reported by Mihailovic et al. [32] who suggested that this might be related to the low repeatability of vessel density measurements for the deep plexus. We and others [19, 20, 32, 33] have also reported decreased SRCP density in TAO patients. The superficial retinal microvasculature is more vulnerable to slight damage of DON because it is located in the nerve fiber and ganglion cell layers [34, 35]. We speculate that the continuous decrease of  SRCP density may be responsible for the transition from non-DON to DON. However, Ye et al. [36] reported that the macular microvascular densities were significantly increased in active TAO patients. This discrepancy may be due to the fact that participants in Ye et al.'s study were primarily active TAO patients uncomplicated with DON. Previous studies also found that reduced superior ophthalmic vein flow velocity and orbital venous congestion were the most important characteristics of orbital blood flow in TAO patients [37, 38]. Previous studies also reported that choroidal blood flow was significantly different in TAO patients compared with control subjects [39, 40]. Taken together, these reports indicate that the abnormal hemodynamic state may play an important role in the onset and progression of DON.

Retina thinning has also been suggested to correlate with visual impairments in TAO patients [19–21]. This study showed that GCCL thickness, which includes the thickness of the nerve fiber layer, ganglion cell layer and inner plexiform layer, was significantly decreased in the DON group. Nevertheless, we found that there was no significant difference in GCCL thickness between the control and non-DON groups. Using spectral-domain OCT, Wu et al. [19] revealed that the GCCL thickness around the macula was significantly decreased in TAO patients with or without DON. Mechanical compression, ischemia, secondary increase in IOP as well as intraorbital inflammation are considered contributing factors for retinal ganglion cells atrophy in TAO [41]. Thus, we hypothesize that retinal ganglion cells atrophy may have occurred in the non-DON group although this subtle degeneration of retinal structure was difficult for OCTA to detect.

We further analyzed the relationship between GCCL thickness and inner retinal microvascular density. Our results demonstrated a significant correlation between the GCCL thickness and SRCP density in patients with TAO. Nevertheless, the causal relationship between GCCL thinning and pathological changes in the superficial retinal microvasculature is unclear. Reduced SRCP density is correlated with reduced oxygen and nutrient availability for the retinal ganglion cells and may contribute to the decreased GCCL thickness [34, 35]. Another possibility is that degeneration of retinal ganglion cells may result in lower oxygen demand from the SRCP [42], and the thinning of the SRCP may be secondary to the thinning of the GCCL.

Interestingly, the stepwise multi-regression analysis showed that decreased SRCP density was the most important factor related to AULCSF deficit in TAO patients. Moreover, we also found that the AULCSF loss was correlated with decreased SRCP density even after adjustment for SE, GCCL thickness and MI. Similar results were also reported by Wu et al. [19, 42] who found that VA was negatively correlated with SRCP density. Neural activity of the retina depends on local blood flow, and macular ischemia is related to visual function [43]. The SRCP is important for oxygen and nutritional support of the retinal ganglion cells. Therefore, hypoperfusion of the superficial retinal microvasculature may be related to the loss of retinal ganglion cells, resulting in visual impairments in TAO. These findings can help us develop a visual protection strategy for patients with early DON.

In this study, we found that decreased SRCP density was the most important factor associated with the early vision loss in TAO. This is probably because our enrolled patients did not show significant orbital apex crowding and most of them had normal IOP (9.3 to 24.8 mmHg) and lower CAS values (0 to 3). However, we still acknowledge the role of mechanical compression, evaluated IOP and intraorbital inflammation in visual impairments of DON. For example, Mourits et al. [16] found that the CSF of DON patients with orbital apex crowding decreased at all spatial frequencies and improved after orbital decompression. De Marco et al. [15] reported that TAO patients with ocular hypertension or suspect glaucoma had pronounced CSF loss, particularly in the low spatial frequency range (0.18 to 0.71 cpd), when compared to DON patients. Labonia et al. [6] found that subclinical VF loss was mainly related to TAO clinical activity and they speculated that inflammation of the orbital tissues would have a more detrimental effect on vision loss in patients with more active TAO. We also cannot rule out the contribution of altered tear dynamics to the loss of CSF as TAO patients can have altered tear dynamics if they have severe exophthalmos. Further studies with more diverse patient groups are necessary to verify the exact roles of these risk factors underlying the visual impairments of DON.

Lastly, we conducted ROC curve analysis to determine the best indicator of early DON. We found that classifying non-DON and DON groups from the controls based on the AULCSF was significantly more accurate than using inner retinal microvascular density, GCCL thickness, or a combination of these indicators. However, because we did not enroll moderate to severe DON patients and did not perform a longitudinal study to follow these patients, we were unable to evaluate the role of CSF in predicting the development of DON. Nevertheless, our results suggest that the CSF may be a valuable endpoint for assessing functional vision in non-DON patients and may be useful in the diagnosis of early DON.

Orbital apex crowding can be measured using different parameters, including MI, maximum MR diameter, extraocular muscle (EOM) volumes, superior ophthalmic vein dilatation and intracranial fat prolapse. EOM volumes could provide more information than cross-sectional measurements because it uses data from a wide section of the muscles [44]. However, the volume measurement is time-consuming and difficult to perform in routine clinical practice. It has been shown that maximum MR diameter was equally predictive of compressive optic neuropathy as EOM volumes [45, 46]. A previous study indicated that MI was a convincing index of orbital apex crowding [24], so we used both maximum MR diameter and MI to provide valid assessment of the degree of mechanical compression. Radiological imaging provides direct evidence for apical optic nerve compression. Clinically, CT and magnetic resonance imaging (MRI) are common ways for the evaluation of DON. Compared to CT, MRI allows for superior soft tissue imaging and can be more sensitive in assessing microstructural changes of the optic nerve in DON patients [47, 48]. In future studies, we may use MRI to evaluate apical crowding instead.

It has been reported that the gender ratio of patients with TAO showed a predominance of females over males [49]. Here, more female than male subjects were recruited. Although we saw a higher proportion of female subjects, there was no significant gender ratio difference among the three groups (*P* = 0.752). We analyzed the data at the gender level, and found that the results were consistent with those with all the patients. The subgroup analysis for male patients would suffer from a lower power because the sample size was small, and thus we decided to pool and analyze the data from all the subjects as a whole.

We recognize that this study has some limitations. First, we only included early DON patients with mild visual impairments and no significant apical crowding or relative afferent pupillary deficits. This means that the results of our study may not apply to patients with moderate to severe DON or significant visual impairments. In order to fully understand the functional and structural damage in patients with TAO, future studies with larger sample sizes and a range of DON severity are needed. Second, this study is cross-sectional, so we cannot determine whether CSF loss in TAO patients leads to an increased risk of developing DON. Longitudinal studies that track CSF loss over different stages of the disease will be necessary to determine the utility of CSF as a clinical endpoint for DON.

## Conclusions

In conclusion, the CSF was significantly reduced even in TAO patients without other clinical signs of DON and could be a sensitive and effective endpoint for assessing visual impairments of TAO patients at risk for DON. In addition, we found that reduced CSF was significantly correlated with decreased SRCP density in the early stage of DON, suggesting that reduced microvascular density around the macula may be one of the most important factors related to early visual impairments before the appearance of obvious orbital apex crowding in TAO.

## Data Availability

The datasets used and/or analyzed during the current study are available from the corresponding author on reasonable request.

## Abbreviations

TAO: Thyroid-associated ophthalmopathy
DON: Dysthyroid optic neuropathy
VA: Visual acuity
BCVA: Best-corrected visual acuity
VF: Visual field
IOP: Intraocular pressure
CSF: Contrast sensitivity function
qCSF: Quick CSF
AULCSF: Area under the log contrast sensitivity function
cpd: Cycle per degree
OCT: Optical coherence tomography
OCTA: Optical coherence tomography angiography
GCCL: Ganglion cell complex layer
SRCP: Superficial retinal capillary plexus
DRCP: Deep retinal capillary plexus
CAS: Clinical activity score
MD: Mean deviation
SE: Spherical equivalent refractive error
D: Diopter
CT: Computed tomography
MRI: Magnetic resonance imaging
MI: Muscle index
MR: Medial rectus
EOM: Extraocular muscle
ROC: Receiver operating characteristic
AUC: Areas under the receiver operating characteristic curve
